# Population attributable fractions for risk factors and disability burden of dementia in Jiangxi Province, China: a cross-sectional study

**DOI:** 10.1186/s12877-022-03507-4

**Published:** 2022-10-21

**Authors:** Yuhang Wu, Huilie Zheng, Fenfei Xu, Jin Guo, Zhitao Liu, Shengwei Wang, Xiaoyun Chen, Yu Cao, Wei Zou, Songbo Hu

**Affiliations:** 1 Jiangxi Province Key Laboratory of Preventive Medicine, School of Public Health, Nanchang University, Nanchang, 330006 Jiangxi China; 2 Department of Epidemiology and Health Statistics, Xiangya School of Public Health, Central South University, Changsha, China; 3 Health Development Center of Jiangxi Province, Nanchang, China

**Keywords:** Dementia, population attributable fractions, years lived with disability, Chinese older people

## Abstract

**Background:**

In view of the fact that there is no effective treatment for dementia, the number of years that dementia patients have to live with dementia will gradually increase for the rest of their lives, and the disability loss caused by dementia will increase. It is urgent to study the influence of risk factors on dementia by making use of the potential of prevention. The purpose of this study is to quantify the burden of dementia disability attributable to risk factors by assessing the population attributable fractions (PAFs) in Jiangxi Province, which is one of the regions of moderate aging process of China.

**Methods:**

The prevalence data of nine risk factors were obtained through the Sixth National Health Service Survey in 2018, which covered 2713 older people. Levin’s formula was used to calculate the PAF for each risk factor for dementia. We adjusted the PAF for communality between risk factors, and used these values to calculate overall weighted PAFs and the years lived with disability (YLDs), which were attributable to nine risk factors.

**Results:**

The number of dementia cases and their proportions that can theoretically be prevented by nine identified risk factors were 111636 (99595-120877) and 66.8% (59.6-72.3), respectively. The total YLDs of dementia were estimated to be 61136 (46463-78369) (males: 36434 [24100-49330], females: 23956 [14716-34589]). Physical inactivity (11639 [8845-14920]), low social contact (9324 [7086-11952]), and hearing loss (5668 [4307-7265] were the top three contributors to dementia.

**Conclusions:**

The moderate aging areas represented by Jiangxi Province have great potential in the prevention of dementia. Targeted interventions and management of risk factors can effectively reduce the disability burden of dementia.

**Supplementary Information:**

The online version contains supplementary material available at 10.1186/s12877-022-03507-4.

## Introduction

According to the Global Burden of Disease (GBD) Study 2016 [1] , the global number of individuals who lived with dementia was 43.8 million in 2016, more females than males had dementia, and the number of prevalent cases of dementia more than doubled from 1990 to 2016. It is estimated that there are 249.49 million people over the age of 60 in China, of whom 15.07 million have dementia [2]. With the ageing of Chinese society, the prevalence of dementia is showing an upward trend [1]. Despite improved access to treatment and health services, inadequate diagnosis and management of dementia are still common [3].

As the country with the most dementia patients in the world, China has a heavy burden on its public and medical system [3]. In view of the fact that there is no effective treatment for dementia, the number of disability-adjusted life years (DALYs) increased from 1.85 million in 1990 to 5.98 million in 2019 in China [4]. Dementia is the main cause of disability for older people [5, 6]. The disability losses caused by dementia are accumulating rapidly, and the number of years lived with disability (YLDs) increased by 308.7% in China over the past three decades [4]. These losses may increase with the progression of the ageing process [1]. Further, a multicenter survey of 3,098 dementia patients in China reported that the total cost of care was $19144 per person per year, and the annual total costs associated with dementia will be $507.49 billion in 2030, $1.00 trillion in 2040, and $1.89 trillion in 2050 in China [7]. There is growing evidence that strengthening the management and monitoring of risk factors for dementia is effective [8–10]. If lifestyle and other interventions are implemented effectively, they may help delay the onset of dementia and reduce the number of dementia patients in the future [9]. A predictive model estimates that by 2050, delaying the onset of Alzheimer’s disease by one year will reduce the total number of cases of Alzheimer’s patients over 60 years old worldwide by 11% [11].

The prevention potential of dementia is huge, especially for China, which has a large older population. The key to preventing dementia is a reliable analysis of risk factors. At present, Liu et al. [12] reported population attributable fractions (PAFs) of seven main risk factors and their effect on dementia for the first time in China. The PAF is defined as the percentage of a disease that will be eliminated if a certain risk factor is eliminated. The value of the PAF depends on the prevalence of the risk factor and the relative risk of the risk factor and the disease. The advantage of the single risk factor approach is that it highlights the potential of a single risk factor. However, some risk factors may be intertwined. For example, lack of physical activity can easily cause obesity, and these are all related to education. Therefore, the reported combined PAF is likely to be overestimated [8]. The first adjusted PAF estimate for dementia related to China was calculated by the 10/66 Dementia Research Group study conducted in 2004–2006, but the use of relative risk (RR) comes from systematic reviews and meta-analyses from predominantly Western countries [13]. A recent study based on the China Multicenter Dementia Survey (CMDS) evaluated the PAFs of eight potentially modifiable risk factors for dementia in China and revealed that China had a unique risk profile owing to its rural–urban disparity [14]. In these studies, the participants were mainly from Beijing municipality [13], Hubei Province [14], Hebei Province [14], and Tianjin municipality [14], with a relatively older population, while the share of people aged 60 and over in many regions (including Jiangxi Province) was lower than the national average [15]. Health problems and access to health care providers and services remain heterogeneous between the provinces in mainland China [16]. Close examination of population health metrics at the province level will be crucial to developing evidence-based policies and achieving the Healthy China 2030 goals. To the best of our knowledge, there is currently very limited evidence to support dementia prevention strategies in regions with moderate ageing population in China.

In this study, we estimated the PAFs of nine potentially modifiable risk factors for dementia in Jiangxi Province, a region with moderate aging population in China, and YLDs were used as an indicator to quantify the burden of dementia disability attributable to the risk factors by assessing the PAF.

## Methods

### Data

The study was an analysis of cross-sectional data obtained from the Sixth National Health Service Survey in 2018 conducted by the National Health Commission of the People’s Republic of China comprehensive one-phase prevalence surveys, given that this survey reported data on the nine identified potentially modifiable risk factors for dementia. The details of this survey have been described previously [17]. Donghu, Zhanggong, Yuanzhou, Shanggao, Gaoan, and Poyang were selected as sample counties (cities, districts) representing the overall situation in Jiangxi Province by multistage stratified cluster random sampling. The investigators asked all participants of the survey household one by one the questionnaire items after being uniformly trained by the Statistics Information Center of the Health and Family Planning Commission. In this questionnaire, the identification of dementia was described in the question of whether the participant had been diagnosed with dementia by doctors. There were two options for participants to choose from. If they answered “yes”, they were considered to have dementia, and if they answered “no”, they were not considered to have dementia. Additional assistance was given to those who were unable to understand an overview of dementia by trained investigators. Before the participants chose the answers based on their dementia experience, the investigators read the questions and provided the participants or their first-degree relatives or primary caregivers with appropriate explanations in accordance with the International Classification of Diseases 10th revision (ICD-10) diagnostic criteria for dementia. Overall, those who answered “yes” were patients who had been diagnosed with dementia at the hospital. Since the survey method was to fill out the questionnaire face to face, the number of subjects lost due to rejection or noncooperation was reduced, and the response rate was improved.

### PAF

A Lancet Commission Report [9] suggested that modifiable risk factors including hearing loss, education, smoking, depression, physical inactivity, social isolation, hypertension, diabetes, and obesity could account for as much as 35% of the dementia burden. In this study, all the risk factors reported were selected except depression. The definition and odds ratio (OR) of each risk factor are presented in the Supplemental Table 1. The OR value for individual risk factors according to the definition of each risk factor comes from previously published studies in the Chinese population [2, 14, 18, 19]. Except for obesity, which was measured by body mass index (BMI), the other factors were all reported by participants or their first-degree relatives or primary caregivers. The questionnaire items and their possible responses are described in the Supplemental Table 2. Since there were no missing data for all variables in the sample, the prevalence of each risk factor can be directly estimated.

The PAF for each risk factor was calculated using Levin’s formula:

$$PAF=\frac{P\times \left( OR-1\right)}{1+P\times \left( OR-1\right)}$$

where P represents the population prevalence of each factor and OR represents the corresponding odds ratio.

However, people may have multiple risk factors at the same time. Therefore, it was very important to consider the communality and calculate the weighted PAF. The communality was calculated as the sum of the square of all factor loadings via principal components analysis of the inter-risk-factor correlation matrix [8, 9, 13]. Each individual risk factor’s PAF was weighted according to its communality using the formula:

$$weight(w)=1- communality$$

Weighting was included in the calculation of overall the PAF using the formula [13]:

$$PAF=1-\left[\left(1-{w}_1\times {PAF}_1\right)\left(1-{w}_2\times {PAF}_2\right)\left(1-{w}_3\times {PAF}_3\right)\dots \right]$$

### YLD

We mainly referred to the general methods of the GBD study [1] to calculate the YLD of dementia in Jiangxi Province. First, the prevalence of dementia was divided into three severity categories (mild; moderate; severe) based on the health status lay description and disability weight of dementia according to the GBD Study 2019 [20], and more information was presented in Supplemental Table 3. Second, we multiplied the prevalence at each severity level by the corresponding disability weight and summed them. Third, we corrected for comorbidity, assigning the adjusted PAF to each risk factor. The definition of dementia in the ICD-10 requires a cognitive impairment sufficient to impair personal activities of daily living (ADL) [21], and the application of ADL to assessment of disability in dementia could be observed in the GBD study and other measuring tools [22]. Therefore, we obtained a series of variables related to ADL provided by their first-degree relatives or primary caregivers in the original data to identify disability in people with dementia. These variables were referred to two dimensions (self-care and usual activities) of the EuroQol-5-dimensions-5 levels (EQ-5D-5L), which included the eight basic activities of eating, dressing, bathing, getting in and out of bed, going to the toilet, controlling defecation, doing housework, and managing money and property in the individual’s daily life. Those who could not complete one or more items were classified as patients with severe dementia, and those who could complete all items without difficulty were classified as patients with mild dementia. Others were classified as moderate dementia. In recent years, assessment of ADL has been regarded as equally effective in appraising dementia severity, and has been applied in many previous studies [23, 24].

The calculation formula of YLD was as follows:

$$YLD=\sum W\times {P}^{\prime}\times N$$

where *W* represents the disability weight of the severity of dementia, *P*^′^ represents the estimated value of the prevalence of dementia, and *N* represents the population of Jiangxi Province in 2018.

Then the YLD for each risk factor was calculated:

$${YLD}_i={PAF}_i\times YLD$$

We also reported the estimated YLDs of each risk factor with the 95% uncertainty interval (UI) in this study. The 95% UI was obtained by repeatedly sampling the sample 1000 times, whose upper and lower bounds were derived based on the 2.5^th^ and 97.5^th^ percentiles of the uncertainty distribution.

## Results

We obtained a total of 2713 valid responses from 10,123 participants (aged 60 and above: 2784). The response rate for the dementia survey among people in their 60s was approximately 97%. The reason for the high response rate of the survey was that the survey was only based on the answers to the questionnaire without physical and mental examinations, so that few people were lost to follow-up or rejected. In this survey, a total of 66 older people had dementia, and the prevalence of dementia in Jiangxi Province in 2018 was 2.4% (Table 1). Epidemic features of dementia are presented in Table 2. A total of 38 males and 28 females were diagnosed with dementia. More cases of dementia were found in urban areas (53.0%). The majority of dementia cases occurred in older people aged 70 years and older, who accounted for 78.8% (70-79: 39.4%, ≥80: 39.4%). The lowest proportion was in the 60-69 years age group, who accounted for 21.2%.

**Table 1:** Prevalence of dementia in Jiangxi Province in 2018

Counties (cities, districts)	participants	The number of dementias	Prevalence (%)
Donghu	547	8	1.5
Gaoan	423	14	3.3
Poyang	336	9	2.7
Shanggao	462	8	1.7
Yuanzhou	522	10	1.9
Zhanggong	423	17	4.0
Total	2713	66	2.4

**Table 2:** Epidemic features of dementia in Jiangxi Province in 2018

Epidemic features	Mild dementia (%)	Moderate dementia (%)	Severe dementia (%)	Total (%)
**No. of dementia**	12 (18.2)	13 (19.7)	41 (62.1)	66 (100)
**Sex**				
males	6 (9.1)	6 (9.1)	26 (39.4)	38 (57.6)
Females	6 (9.1)	7 (10.6)	15 (22.7)	28 (42.4)
**Address**				
Urban	6 (9.1)	4 (6.1)	25 (37.9)	35 (53.0)
Rural	6 (9.1)	9 (13.6)	16 (24.2)	31 (47.0)
**Age ranges (years)**				
60-69	2 (3.0)	4 (6.1)	8 (12.1)	14 (21.2)
70-79	7 (10.6)	7 (10.6)	12 (18.2)	26 (39.4)
≥80	3 (4.6)	2 (3.0)	21 (31.8)	26 (39.4)

The number of exposures and prevalence, communality, weighted PAFs and number of attributable cases of nine risk factors associated with dementia for Jiangxi Province in China in 2018 are presented in Table 3. Low social contact, physical inactivity, hypertension and hearing loss were the top four contributing factors, at 77.6, 54.2, 34.4 and 33.8%, respectively. Obesity had the lowest contribution (1.6%). In Jiangxi, the number of dementia cases and their proportions that can theoretically be prevented by 9 identified risk factors were 111636 (99595-120877) and 66.8% (59.6-72.3), respectively. Physical inactivity (19.0% [18.8-19.1]) and low social contact (15.3% [14.2-15.8]) were the top two fraction contributors to dementia, followed by hearing loss (9.3% [8.2-10.0]), hypertension (7.2% [6.3-7.3]), low education (6.8% [5.7-7.7]) and smoking (5.2% [4.5-5.3]). Having no spouse (2.5% [1.4-4.3]), diabetes (0.8% [0.3-1.2]) and obesity (0.8% [0.3-1.6]) showed relatively small effects on dementia.

**Table 3:** Estimates for population attributable fractions (PAFs) and the number of attributable cases in Jiangxi Province in 2018

Risk factors	Number of exposures (Prevalence) (%)	Communality (%)	Weighted PAF (95% CI) (%)	Number of attributable cases (95% CI) ^a^
Low education	714 (26.3)	64	6.8 (5.7-7.7)	11391 (9450-12837)
No spouse	601 (22.2)	61	2.5 (1.4-4.3)	4140 (2311-7194)
Smoking	617 (22.7)	56	5.2 (4.5-5.3)	8612 (7454-8825)
Physical inactivity	1471 (54.2)	57	19.0 (18.8-19.1)	31824 (31427-31928)
Obesity	43 (1.6)	34	0.8 (0.3-1.6)	1344 (431-2666)
Low social contact	2104 (77.6)	60	15.3 (14.2-15.8)	25493 (23754-26421)
Hearing loss	916 (33.8)	40	9.3 (8.2-10.0)	15497 (13733-16742)
Hypertension	933 (34.4)	47	7.2 (6.3-7.3)	12001 (10550-12209)
Diabetes	243 (9.0)	59	0.8 (0.3-1.2)	1335 (485-2054)
Overall weighted PAF			66.8 (59.6-72.3)	111636 (99595-120877)

^a^Number of attributable cases of dementia in Jiangxi in 2018 = 167164.

**Fig. 1:**
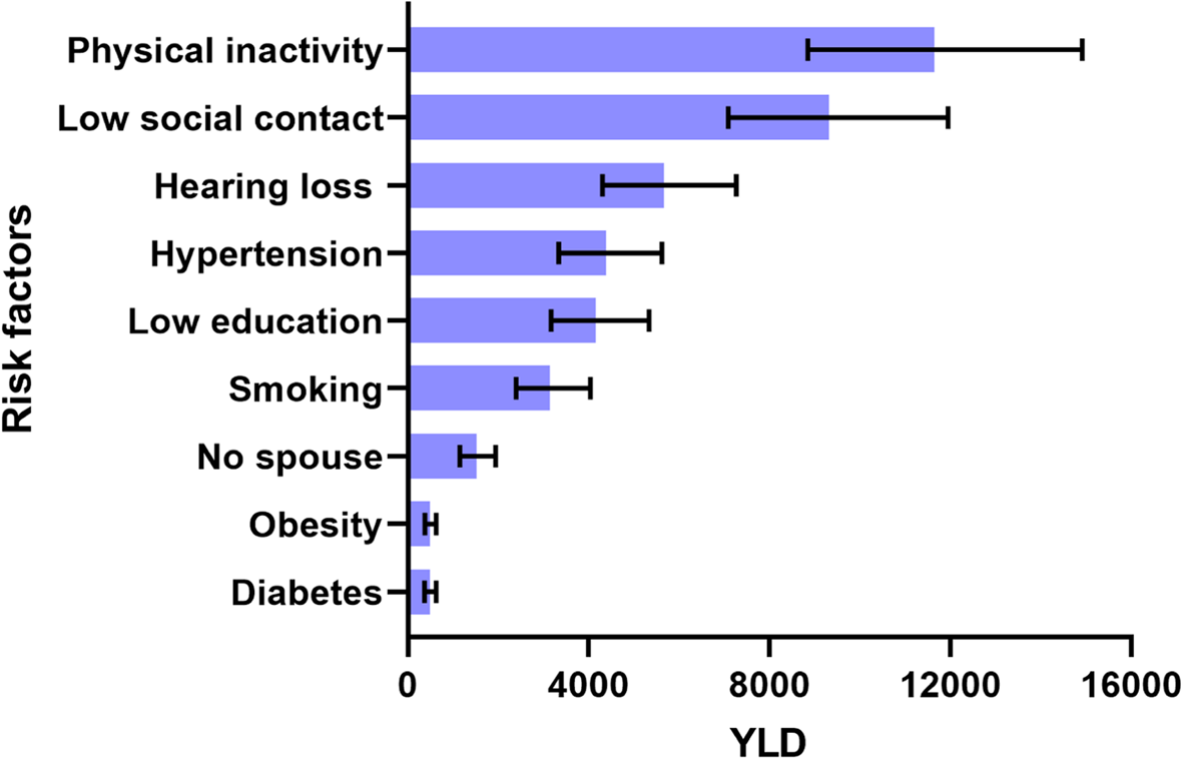
Estimates for years lived with disability (YLDs) in Jiangxi Province in 2018

Figure 1 shows the estimates of YLDs of nine risk factors leading to dementia in Jiangxi in 2018. The total YLDs of dementia in Jiangxi Province in 2018 was estimated to be 61136 (46463-78369) (males: 36434 [24100-49330], females: 23956 [14716-34589]), of which the YLDs of the nine risk factors leading to dementia was 40828 (31029-52337). The top three risk factors were physical inactivity (11639 [8845-14920]), low social contact (9324 [7086-11952]), hearing loss (5668 [4307-7265].

## Discussion

The findings of this study indicated that after adjusting for the nonindependence of risk factors, more than 60% of dementia cases in Jiangxi Province in China in 2018 were associated with the nine potentially modifiable risk factors assessed here. Among the nine factors, the PAFs of the five classical risk factors (low education, physical inactivity, low social contact, hearing loss, and diabetes) for dementia were incorporated in previous studies but were higher in our study (51.2%) than CMDS estimates in China (42.8%); the PAFs of the eight classical risk factors (hearing loss, low education, smoking, physical inactivity, low social contact, hypertension, diabetes, and obesity) for dementia were higher in our study (64.3%) than 10/66 estimates in China (39.0%), indicating the especially higher potential for dementia prevention in moderate aging regions of China. The burden of disability due to dementia in Jiangxi Province in 2018 was heavy, of which physical inactivity, low social contact, and hearing loss were the three most important factors. As a quantitative study to estimate the burden of dementia caused by risk factors, we evaluated the burden of dementia caused by nine risk factors using YLDs as an index. It may provide an opportunity for the general public and dementia patients to consider better management and prevention strategies with a targeted understanding, thereby reducing the risk burden. There was a higher total number of YLDs attributable to the 9 risk factors in males than in females.

The PAF of low education level (lower than primary school) was 6.8%, which was lower than previous studies on the Chinese population [12–14]. Mechanically, it is believed that lifelong education and mental stimulation can help build a “cognitive reserve” to reduce the risk of dementia, which enables individuals to continue to work at a “normal level” even though they experience neurodegenerative changes [25]. Historically, around the 1970s in China, Jiangxi Province experienced a unique period in which many young people did not have the opportunity to receive adequate education. However, with the popularization and improvement of education, disability burden of dementia due to low levels of education will be effectively controlled. Physical inactivity was the largest PAF (19.0%) contributor to dementia, resulting in the greatest number of years of disability and life lost caused by dementia among nine risk factors, which was higher than CMDS estimates (13.2%) and 10/66 estimates (5.8%) [13, 14]. Physical exercise modulates amyloid β turnover, inflammation, the synthesis and release of neurotrophic factor, and cerebral blood flow [26]. These positive effects are effective in improving the progression of age-related neurodegeneration, and the benefits may accumulate throughout a person’s life. Physical inactivity was more common in Jiangxi Province than in other parts of China [14], possibly due to a lack of consensus on health and cultural promotion for older people, which hinders the promotion of health plans and appropriate physical activity programs. It was calculated by our model that low social contact was the second major reason for the heavy burden of dementia, which was caused by the higher prevalence and higher OR. Social conditions in China have changed significantly over the past two decades, and nearly 80% of the older people lack social contact in Jiangxi Province, which is higher than the overall prevalence in China [14]. Social contact indicates a social connection with friends, not relatives. Compared with relatives, keeping in touch with friends can bring more happiness and less pressure, because friends reflect personal choices [27]. More frequent contact will bring higher cognitive reserves, while higher cognitive capabilities can prevent dementia that may occur later [28]. Pathways associated with these connections include inflammatory responses in the brain, behavioral or neural plasticity, and the occupation of cognitive resources available for creative adaptations [29].

The recognition of hearing loss as a risk factor for dementia was relatively new, although some studies [9, 13, 14] have previously reported on the calculation of its PAF. The estimated PAF for hearing loss in our research (9.3%) was between the CMDS estimates (11.9%) and 10/66 estimates (3.9%), which was mainly based on data from developed countries and regions. The mechanism of hearing loss induced dementia remains unclear. Timothy evaluated candidate brain bases for this relationship, including a common pathology affecting the ascending auditory pathway and multimodal cortex, depletion of cognitive reserve due to an impoverished listening environment, and the occupation of cognitive resources when listening in difficult conditions [30]. Early implementation of hearing protection will help to reduce the burden of this potential risk factor for dementia. Preliminary evidence suggests that the use of hearing aids can reduce the risk of dementia due to hearing loss [13]. Compared with the 10/66 estimation for China, hypertension had a greater contribution to dementia in this study, and the burden of disease due to hypertension was also larger. The OR of hypertension used in the present study was higher than that for other countries [8, 31]. In addition to inducing cognitive impairment through structural and functional impairment of the cerebral blood vessels [32], hypertension also has a direct impact on the functions of the central nervous system through changes in the cerebral renin–angiotensin system [33]. The prevalence of hypertension in China was gradually increasing and younger, whereas the awareness and treatment rate of Chinese hypertension patients did not increase [12, 34]. In this study, the prevalence of hypertension was estimated to include older patients with hypertension who were diagnosed by doctors but not those who were not diagnosed or reported, suggesting that our results may underestimate the impact of hypertension on dementia. It is worth noting that the PAF of smoking for dementia based on the OR in the CMDS was higher than the 10/66 estimates in China, and most of the burden of dementia caused by smoking may come from males in Jiangxi Province. Even in old age, the prevalence of smoking among males was still as high as 43.3% (females: 3.6%). The most likely mechanism of the association between smoking and dementia is cardiovascular disease [35]. Atherosclerosis and cerebrovascular diseases caused by smoking in turn increase the risk of dementia [36]. Cigarettes also contain neurotoxins, which also increase the risk of dementia [37]. China is one of the largest tobacco consumers in the world, although interventions to reduce smoking are already being implemented throughout China (including Jiangxi Province). The public’s awareness of smoking bans and tobacco control is increasing, but this has not effectively reduced the smoking rate. Having no spouse, diabetes and obesity bear relatively little burden on dementia. Compared with people living with spouses, widowed, divorced or unmarried people had a particularly increased risk of dementia [18, 38], which was similar to CMDS estimates in China. Living with a partner may imply cognitive and social challenges that can protect against cognitive impairment in later life [38]. In addition, having no spouse might result in loneliness and less communication or mutual assistance [39]. Adipose tissue produces several substances that are important in metabolism (adipokines) and inflammation (cytokines) and are correlated with insulin resistance and hyperinsulinemia [40]. Similarly, due to unhealthy eating habits and lack of awareness of diabetes prevention, China has witnessed one of the fastest rising prevalence rates of diabetes in the world [41]. There were still quite a few diabetic patients who were not diagnosed in this study, which was similar to the prevalence of hypertension. More work should be done in the diagnosis and treatment of diabetes to further reduce the risk of dementia development and reduce the burden of dementia caused by diabetes. Compared with the 10/66 estimation for China [13], obesity was less prevalent in our survey, but the change to a Western diet together with physical inactivity may likely increase the prevalence of obesity in the future.

There are some bidirectional associations between some risk factors and dementia. Dementia could in turn affect exposure levels of physical activity, and people with dementia are less physically active than their cognitively healthy counterparts. Reasons for this are multifaceted, and are thought to be social, psychological, and physiological [42]. Moreover, social dysfunction is a part of dementia. As the severity of dementia increases, the time spent with others decreases, and these changes have been described in the prodromal phase [43]. Therefore, it may be a consequence rather than a cause of dementia, which suggests that the estimates of PAF for these risk factors may be overestimated in our study. In other words, it may not achieve the expected potential to prevent dementia only by addressing these risk factors.

Our study has some limitations. First, the dementia status of the participants was self-reported in this study, although those with dementia were diagnosed by doctors. However, it must be acknowledged that the prevalence of dementia we estimated may be underestimated by ignoring individuals who may have dementia but were not diagnosed in hospitals. Some information about the risk factors was also self-reported, which may lead to some information being misclassified due to lack of standardization. Second, this study did not include cohort studies on the risks associated with the assessed factors. The estimate of the OR relied on secondary data, which was determined by published studies involving Chinese populations. Third, only ADL was considered to identify dementia disability in this study, while other characteristics of dementia described by the GBD study, such as memory loss and cognitive impairment, were ignored. However, assessment of ADL has been regarded as equally effective in appraising dementia severity, which is supported by some studies [23, 24]. Furthermore, the information was obtained through questionnaires rather than direct measurement tools, and our results may be affected by some measurement bias. In future research, it remains more appropriate to combine information from multiple dimensions such as care needs, ADL, and cognitive impairment to improve the measurement of dementia disability.

## Conclusions

With the increase in life expectancy, the process of ageing is accelerating, and dementia will become one of the biggest public health challenges in the world. Over 60% of dementia cases may be attributed to the nine potentially modifiable risk factors included in our study. Harnessing the potential of prevention is an urgent priority. Addressing physical inactivity, low social contact, hearing loss, and hypertension may be initial goals for policy-makers to develop dementia prevention strategies. The government and policy-makers may undertake appropriate costs to delay or prevent dementia cases to reduce the burden of dementia. Even for a small group of people, delaying dementia by a few years would be a huge achievement. Throughout the course of life, early public health measures can be taken not only to prevent premature death but also to promote a healthier life for older people.

## Supplementary Information

**Additional file 1: Supplemental Table 1.** Definitions and odds ratio (OR) used for each of the risk factors.

**Additional file 2: Supplemental Table 2.** The questionnaires and answer options for variables.

**Additional file 3: Supplemental Table 3.** GBD 2019 sequelae, health states, health state lay descriptions, and disability weights.

## Data Availability

The study used data from the Sixth National Health Services Survey (NHSS, 2018) in Jiangxi, the raw case data is closed to public access because it involves sensitive information of the survey respondents, but various statistical on the raw data are publicly available, for details, please refer to the book, An Analysis Report of National Health Services Survey in China, 2018 (Center for Health Statistical and Information. An Analysis Report of National Health Services Survey in China, 2018. People’s Medical Publishing House; 2021.). We obtained administrative permission to access and use the raw data.

## Abbreviations

PAF: Population attributable fractions
YLDs: Years lived with disability
DALYs: Disability-adjusted life years
GBD: The Global Burden of Disease study
RR: Relative risk
OR: Odd ratio
ICD-10: The International Classification of Diseases 10th revision
BMI: Body mass index
ADL: Activities of daily living
EQ-5D-5L: EuroQol-5-dimensions-5 levels
95% CI: 95% uncertainty interval
