# Supplementary material for: Population attributable fractions for risk factors and disability burden of dementia in Jiangxi Province, China: a cross-sectional study
Source: BMC Geriatr. 2022 Oct 21;22:811. doi: 10.1186/s12877-022-03507-4 (PMC9587554; doi:10.1186/s12877-022-03507-4)
Supplement: Supplementary file 1 — Additional file 1: Supplemental Table 1. Definitions and odds ratio (OR) used for each of the risk factors. [file 12877_2022_3507_MOESM1_ESM.docx]

Supplemental table 1. Definitions and odds ratio (OR) used for each of the risk factors

| Risk factors | Definition | Odds ratio |
| --- | --- | --- |
| Low education | Not receiving more than primary education in early life | 2.1 (1.8-2.4) |
| No spouse | Self-reported current unmarried, widowed or divorced status | 1.4 (1.2-1.8) |
| Smoking | Self-reported smoking in later life (Excludes smoking but has quit) | 1.9 (1.7-2.0) |
| Physical inactivity | Self-reported that the number of conscious physical exercises (morning exercises, break exercises, physical education classes, extracurricular sports classes, work exercises, square dancing, walking exercises, walks, running) per week is less than once | 4.1 (3.6-4.6) |
| Obesity | A body mass index (BMI) measurement of more than 30Kg/m^2^ | 2.7 (1.5-4.7) |
| Low social contact | Self-reported participation in social activities (community patrols, caring for other people, environmental protection, dispute mediation, accompanying chat, volunteer service, child care, etc.) less than once | 2.3 (2.0-2.6) |
| Hearing loss | Self-reported hearing impairment | 2.3 (2.0-2.6) |
| Hypertension | Diagnosed with hypertension by doctor | 1.9 (1.7-2.0) |
| Diabetes | Diagnosed with diabetes by doctor | 1.3 (1.1-1.5) |
