# Supplementary material for: Population attributable fractions for risk factors and disability burden of dementia in Jiangxi Province, China: a cross-sectional study
Source: BMC Geriatr. 2022 Oct 21;22:811. doi: 10.1186/s12877-022-03507-4 (PMC9587554; doi:10.1186/s12877-022-03507-4)
Supplement: Supplementary file 2 — Additional file 2: Supplemental Table 2. The questionnaires and answer options for variables. [file 12877_2022_3507_MOESM2_ESM.docx]

Supplemental table 2. The questionnaires and answer options for variables

| Variable name | Answer |
| --- | --- |
| Educational level | 1= Not receiving more than primary education  2= Primary education or above |
| Marital status | 1= Unmarried 2= Married 3= Widowed 4= Divorced |
| Current smoking status | 1= Yes 2= Quit smoking 3= No |
| How many times per week have you consciously exercised in the last 30 days? | 1= Once a week or more 2= Less than once a week |
| Have you ever been diagnosed with hypertension? | 1= Yes 2= No |
| Have you ever been diagnosed with diabetes? | 1= Yes 2= No |
| Have you ever been involved in any social activities? | 1= Yes 2= No |
| Which of the following conditions did you have in terms of hearing in the last 6 months? | 1= I can clearly hear what others say 2= I can't hear clearly and need someone to raise the volume |
