# Supplementary material for: Population attributable fractions for risk factors and disability burden of dementia in Jiangxi Province, China: a cross-sectional study
Source: BMC Geriatr. 2022 Oct 21;22:811. doi: 10.1186/s12877-022-03507-4 (PMC9587554; doi:10.1186/s12877-022-03507-4)
Supplement: Supplementary file 3 — Additional file 3: Supplemental Table 3. GBD 2019 sequelae, health states, health state lay descriptions, and disability weights. [file 12877_2022_3507_MOESM3_ESM.docx]

Supplemental table 3. GBD 2019 sequelae, health states, health state lay descriptions, and disability weights

| Sequela | Health state name | Health state lay description | Disability Weight |
| --- | --- | --- | --- |
| Mild Alzheimer's disease and other dementias | Dementia, mild | has some trouble remembering recent events, and finds it hard to concentrate and make decisions and plans. | 0.069 (0.046-0.099) |
| Moderate Alzheimer's disease and other dementias | Dementia, moderate | has memory problems and confusion, feels disoriented, at times hears voices that are not real, and needs help with some daily activities. | 0.377 (0.252-0.508) |
| Severe Alzheimer's disease and other dementias | Dementia, severe | has complete memory loss; no longer recognizes close family members; and requires help with all daily activities. | 0.449 (0.304-0.595) |
